# Ascorbate maintains a low plasma oxygen level

**DOI:** 10.1038/s41598-020-67778-w

**Published:** 2020-06-30

**Authors:** Louise Injarabian, Marc Scherlinger, Anne Devin, Stéphane Ransac, Jens Lykkesfeldt, Benoit S. Marteyn

**Affiliations:** 1 Université de Strasbourg, CNRS, Architecture Et Réactivité de L’ARN, UPR9002, 67000 Strasbourg, France; 2 Université de Bordeaux, IBGCUMR 5095, 1 rue Camille Saint Saëns, 33077 Bordeaux Cedex, France; 3 UMR-CNRS UMR -5164 Immunoconcept, 146 rue Léon Saignat, 33076 Bordeaux, France; 4 Faculty of Health and Medical Sciences, University Copenhagen, Copenhagen, Denmark; 5 Institut Pasteur, Unité de Pathogenèse des Infections Vasculaires, 28 rue du Dr Roux, 75724 Paris Cedex 15, France; 6 INSERM Unité 1225, 28 rue du Dr Roux, 75724 Paris Cedex 15, France; 7 Institut de Biologie Moléculaire et Cellulaire, 15, rue Descartes, 67000 Strasbourg, France

**Keywords:** Chemical biology, Leukocytes

## Abstract

In human blood, oxygen is mainly transported by red blood cells. Accordingly, the dissolved oxygen level in plasma is expected to be limited, although it has not been quantified yet. Here, by developing dedicated methods and tools, we determined that human plasma pO_2_ = 8.4 mmHg (1.1% O_2_). Oxygen solubility in plasma was believed to be similar to water. Here we reveal that plasma has an additional ascorbate-dependent oxygen-reduction activity. Plasma experimental oxygenation oxidizes ascorbate (49.5 μM in fresh plasma vs < 2 μM in oxidized plasma) and abolishes this capacity, which is restored by ascorbate supplementation. We confirmed these results in vivo, showing that the plasma pO_2_ is significantly higher in ascorbate-deficient guinea pigs (Ascorbate_plasma_ < 2 μM), compared to control (Ascorbate_plasma_ > 15 μM). Plasma low oxygen level preserves the integrity of oxidation-sensitive components such as ubiquinol. Circulating leucocytes are well adapted to these conditions, since the abundance of their mitochondrial network is limited. These results shed a new light on the importance of oxygen exposure on leucocyte biological study, in regards with the reducing conditions they encounter in vivo; but also, on the manipulation of blood products to improve their integrity and potentially improve transfusions’ efficacy.

## Introduction

Blood gases are either dissolved in the plasma or transported by hematies. The solubility of O_2_ is low compared to CO_2_^1^. Only a limited fraction of O_2_ is dissolved in plasma, representing less than 2% of the total blood oxygen content. Arterial pO_2_ equals 75–100 mmHg (9.9–13.1% O_2_) and venous pO_2_ equals 30–50 mmHg (3.9–5.6% O_2_); in theory the blood plasma pO_2_ would be ranged from 0.9 to 3 mmHg (0.1–0.4% O_2_), although it has not been experimentally quantified. Until now, it was considered that the solubility coefficient of O_2_ in plasma was similar in water^2^. The impact of ascorbate (or Vitamin C), a strong reducing molecule, on plasma oxygen level has not yet been investigated, despite of its abundance (50–70 μM^3,4^) and their respective standard redox potentials (*E*′^0^_O2/H2O_ = 0.815 and *E*′^0^_DHA/Ascorbate_ = 0.08)^5^.

In this report, we confirmed experimentally that plasma is poorly oxygenated and revealed that ascorbate contributes to its low oxygenation level, by reducing O_2_. The impact of plasma “physiological hypoxia” on circulating cells’ physiology and plasma components’ stability has been further investigated.

## Results

### Blood plasma is poorly oxygenated

**Figure 1:**
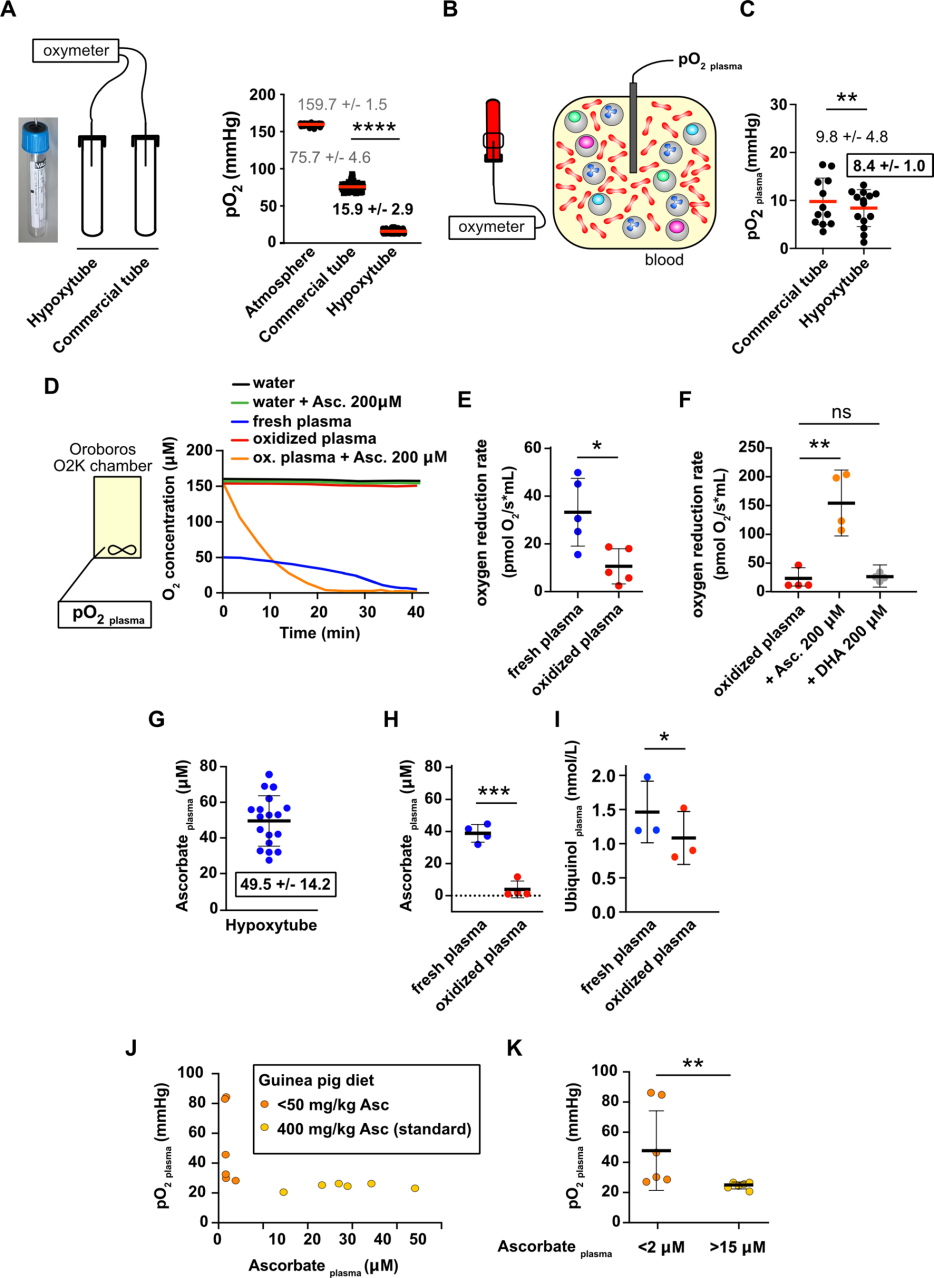
The plasma oxygen level is low, mainly sustained by the ascorbate oxygen. (**A**) Blood collection tubes containing a limited amount of oxygen (Hypoxytubes; picture) have been designed and validated using an oximeter with a microsensor equipped with a steel needle. Commercial tube used as a control was BD Vacutainer K2E (EDTA). Results are expressed as Mean ± S.D.; **** indicates p < 0.0001, n = 20 (tubes). (**B–C**) Plasma oxygen level was directly quantified in whole venous blood collected in commercial tube or Hypoxytube. Plasma pO_2_ quantifications are expressed as Mean ± S.D.; ** indicates p < 0.01, n = 12 individual donors. (**D**) Plasma samples were loaded in closed cuve to record the time-dependent oxygen availability in fresh plasma, oxidized plasma and water, supplemented or not with 200 μM ascorbate (representative experiment). (**E**) Plasma oxygen reduction rates were quantified in fresh or oxidized plasma samples, as described in (**D**). Results are expressed as Mean ± S.D.; * indicates p < 0.05, n = 5 individual samples. (**F**) The impact of oxidized plasma supplementation with ascorbate or dehydroascrobate (DHA) (200 μM) was quantified as described in (**E**). Results are expressed as Mean ± S.D.; ** indicates p < 0.01, n = 4. (**G–H**) Plasma ascorbate concentration in fresh samples was quantified as described in Methods, in blood samples collected in Hypoxytubes (**G**, n = 18 individual samples). The impact of plasma oxygenation on ascorbate concentration is shown in (**H**, n = 4). Results are expressed as Mean ± S.D.; *** indicates p < 0.001. (**I**) Ubiquinol concentration in fresh and oxidized plasma samples was quantified, together with other plasma components (see Figure S1). Results are expressed as Mean ± S.D.; * indicates p < 0.05, n = 3 individual samples. (**J–K**) The impact of plasma ascorbate deficiency on the control of the oxygen level has been investigated in vivo in guinea pigs (**J**–**K**). Plasma ascorbate concentration and pO_2_ was recorded in animals fed standard (400 mg ascorbate/kg) or ascorbate-deficient diet (< 50 mg ascorbate/kg) (**J**). Plasma pO_2_ was average in each group (plasma ascorbate < 2 μM (deficient) and > 15 μM (control)). Results are expressed as Mean ± S.D.; ** indicates p < 0.01, n = 6 animals.

In order to quantify the blood plasma oxygen level, we aimed at avoiding non-physiological oxygenation of samples. All commercially available blood collection tubes contain a significant amount of oxygen, since they are sealed under atmospheric conditions (Fig. 1A, 75.7 ± 4.6 mmHg; 9.9 ± 0.6% O_2_). To avoid blood experimental oxygenation, we designed and produced tubes containing a limited amount of oxygen (Fig. 1A, 15.9 ± 2.9 mmHg; 2.1 ± 0.4% O_2_) hereafter termed Hypoxytube. These non-commercial prototype tubes were produced by the Greiner BioOne company. As opposed to commercial tubes, hypoxic tubes were sealed under a nitrogen atmosphere, hence limiting their oxygen-content. Immediately after blood collection, oxygen level in plasma was quantified with a needle sensor in commercial tubes or Hypoxytubes (Fig. 1B). Plasma pO_2_ was 9.8 ± 4.8 mmHg (1.3 ± 0.6% O_2_) in commercial tubes versus 8.4 ± 1.0 mmHg (1.1 ± 0.1% O_2_) in Hypoxytube (*p* < 0.01): the latest value being the most accurate quantification of the plasma oxygen level (Fig. 1C).

### Ascorbate sustains a plasma oxygen-reduction activity

When fresh plasma pO_2_ was recorded in a closed chamber (Oroboros), a continuous decrease was observed until anoxia was reached (Fig. 1D). This reaction was significantly lower in oxidized plasma or in water (Fig. 1D,E and S1A). These results strongly suggested that an oxidation-sensitive plasma component was supporting its oxygen-reduction capacity. We hypothesized that plasma ascorbate may play a central role in this reaction. Indeed, the supplementation of oxidized plasma with 200 μM ascorbate restored its oxygen reduction activity, not with dehydroascrobate (DHA) (Fig. 1D,F), supporting this hypothesis. However, this reaction does not occur in water (Figure S1A), indicating that the ascorbate-dependent oxygen reduction involved other plasma redox components. Additionally, we demonstrated that plasma ascorbate concentration (49.5 ± 14.2 μM, Fig. 1G) was drastically reduced in oxidized plasma (*p* < 0.001, Fig. 1H). The concentration of ubiquinol, another oxidation-sensitive plasma component was significantly lower in oxidized plasma (*p* < 0.05, Fig. 1I); in association with an increase of its oxidized form (ubiquinone) (Fig. 1I). The concentration of other tested plasma components was unchanged upon plasma oxygenation (salts, proteins or additional oxidation-sensitive components (α-tocopherol, γ-tocopherol, Figure S1B–D).

We confirmed the ascorbate-dependent plasma oxygen reduction capacity in vivo, in guinea pigs, which, like humans, do not synthesize ascorbate. When animals were fed a standard diet, the plasma ascorbate concentration was higher than 15 μM and the plasma pO_2_ controlled at a low level (24.11 ± 2.23 mmHg; 3.17 ± 0.29% O_2_; Fig. 1J–K). These values are higher compared to human plasma, probably due to technical reasons (time between blood collection and pO_2_ measurement). When animals were fed an ascorbate-deficient diet, the plasma ascorbate concentration was lower than 2 μM and the plasma pO_2_ no longer maintained at a low level (50.40 ± 26.32 mmHg; 6.63 ± 3.46% O_2_) (Fig. 1J–K). Altogether these results confirm the in vivo contribution of ascorbate to the maintenance of a low plasma oxygen level.

### Circulating leucocytes sense plasma low-oxygenation—mitochondrial network

**Figure 2:**
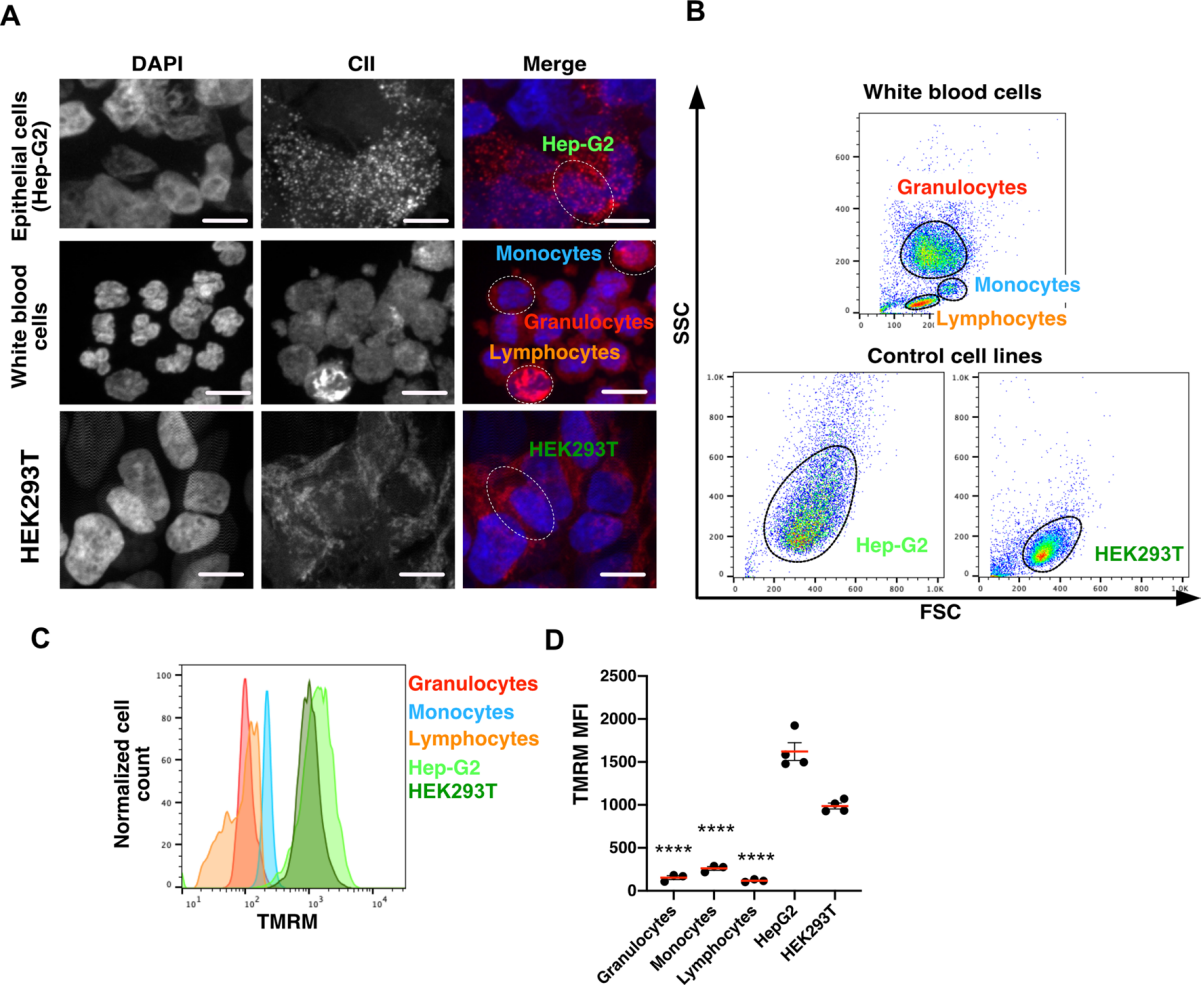
The mitochondria abundance is reduced in circulating leucocytes in low-oxygenated plasma. (**A**) Immunofluorescence staining of white blood cells (WBCs: monocytes, lymphocytes and granulocytes) Hep-G2 cells, and HEK293T cells using anti-CII (mitochondria, red) and DAPI (nuclei, blue). Bars are 10 μm. (**B**) Flow cytometry analysis of WBCs (granulocytes, monocytes and lymphocytes), Hep-G2 and HEK293T. Representation of SSC and FSC profiles. **(C)** TMRM (mitochondria) intensity profiles in granulocytes, monocytes, lymphocytes, Hep-G2 and HEK293T cells (representative experiment) (**D**) Quantification of TMRM mean fluorescence intensity (MFI) in cells described in (**C**). Results are expressed as Mean ± S.D.; **** indicates p < 0.0001 (one-way ANOVA with Tukey’s test, see Tables in Figure S2A), n = 4 independent biological samples.

The adaptation of circulating leucocytes to plasma low oxygen level has not been investigated previously. In other cell-types, it has been reported that under hypoxic conditions, mitochondrial abundance and oxygen consumption is reduced^6–8^. We confirmed by immunofluorescence (Fig. 2A) and flow cytometry (Fig. 2B–D) that, compared to two different cell lines (HEK293T and HEp-G2) cultured under atmospheric conditions (21% O_2_), the mitochondrial abundance of leucocytes (granulocytes, monocytes and lymphocytes) was significantly reduced (ANOVA, Fig. 2C–D). These results strongly suggest that leucocytes evolve under low oxygen conditions in the blood plasma fraction.

Further experiments conducted in vitro at 1% O_*2*_, better reflecting the plasma physiological conditions revealed in this study, will have to be performed to support our conclusions.

## Discussion

We confirm here experimentally that human plasma has a low oxygen level (< 8.4 mmHg, 1.1% O_2_, Fig. 1C) and that ascorbate plays a key role in its maintenance (Fig. 1D). Ascorbate is well described and a strong antioxidant in human plasma, which may either scavenge reactive oxygen species (ROS) or regenerate other plasma antioxidants^9^. Here, we described a physiological and ultimate consequence of the ascorbate reactivity: the dissolved plasma oxygen reduction. Plasma ascorbate is highly susceptible to plasma oxygenation and subsequent oxidation (Fig. 1E). However, our data indicates that ascorbate does not directly react with oxygen (Figure S1A), suggesting that other plasma antioxidants may be involved in its oxygen-depletion capacity. It may be hypothesized that plasma ascorbate acts as a cofactor and increase the oxygen-reduction ability of other plasma components. As an example, ascorbate can bind to human serum albumin, another major antioxidant in the circulation^10^. Further investigations will be required to decipher the overall partners and reactions.

Interestingly, plasma ascorbate concentration is relatively low in plasma (micromolar range, here 50 ± 14 μM, Fig. 1F) compared to human body cells and tissues (millimolar concentrations). Nevertheless, plasma ascorbate concentration is tightly controlled, severe ascorbate deficiency (< 5 μM) is associated with scurvy. We observed in guinea pigs that in these conditions, the plasma low oxygen level was no longer maintained (Fig. 1K). The overall physiological consequences of this regulation defect will have to be further investigated (e.g*.* red blood cell hemoglobin saturation rate, tissue oxygenation efficiency, integrity of other plasma components, leucocyte physiology, among others). In particular, in vitro experiments performed in this study (Fig. 2) were conducted at 0% O_2_ due to technical limitations.

Further experiments will have to be performed at 1.1% O_2_ to even better appreciate the behavior of plasma proteins (stability, oxidation) and leucocyte physiology in the blood circulation.

Plasma ascorbate concentration varies with daily oral intakes but remains controlled at relatively low levels. If 500 mg ascorbic acid/day is sufficient to maintain a physiological plasma level (50 μM), it was shown that 3 g ascorbic acid oral intake every 4 h leads to a maximal plasma ascorbate concentration of only 220 μM^11^. Millimolar plasma ascorbate concentrations may only be reached upon intravenous administration, as currently investigated in the treatment of various cancers, based on the selective cytotoxicity to tumor cells in vitro^12^. Currently, the impact of such high ascorbate concentrations on the plasma pO_2_ is unknown and will have to be determined. In addition, increased plasma ascorbate concentrations have been shown to be associated with an increased production of ascorbate free radicals, a byproduct of self-oxygenation^13^. These free radicals have been proposed to react with transient metal (such as copper and iron), leading to deleterious hydroxyl radical production via the Fenton reaction.

Overall, blood plasma low oxygenation level should be better considered for basic research, diagnostics and therapeutic applications. As an illustration, this statement is critical during blood products collection and preservation prior transfusion to avoid detrimental impact on their quality^14,15^.

## Methods

### Blood collection tubes

Blood samples were collected either in commercial collection tubes (BD Vacutainer K2E (EDTA), ref 368,861) or in Hypoxytubes developed in collaboration with the Greiner Bio One (GBO) company, containing a limited amount of O_2_. (tubes were sealed under a nitrogen atmosphere). Internal pO_2_ was quantified in commercial tubes and in Hypoxytubes using an oximeter with a microsensor equipped with a steel needle (Unisense).

### Blood collection

All participants gave written informed consent and all the study procedures were carried out in accordance with the Declaration of Helsinki principles. Human blood was collected from healthy patients at the ICAReB service of the Pasteur Institut (authorization No. 2020_0120). All donors required to rest in a sitting position for a few minutes before the sampling.

### Cell culture

HEK293T (ATCC CRL-1573) and Hep-G2 (ATCC HB-8065) were cultured in DMEM + 8% SVF. Cells were seeded onto 24-well plates and incubated 24 h at 37 °C at 0% (anoxic cabinet) or 21% O_2_.

White blood cells (WBCs) were purified form whole blood in an anoxic chamber by the addition of a 6% dextran solution (30 min, RT). The WBC-containing supernatant was collected and resuspended in RPMI 1,640 (Thermofisher); remaining red blood cells were eliminated with a lysis buffer.

Cells were fixed in paraformaldehyde (PFA) 3.3% for immunofluorescent labelling or labeled with fluorescent marker for flow cytometry analysis, as previously described^16^.

### Plasma pO_2_ measurement and components’ dosage

Immediately after blood collection, the plasma pO_2_ was measured directly in the blood collection tube using an oximeter with a standardized microsensor equipped with a steel needle (Unisense), as previously described^17^.

Following centrifugation for 5 min at 2,000×*g*, the plasma was acidified with an equal volume of 10% (w/v) metaphosphoric acid (MPA) containing 2 mmol/L of disodium-EDTA. Ascorbate concentration was quantified by high-performance liquid chromatography with coulometric detection, as described previously^18^. Likewise, using high-performance liquid chromatography with coulometric detection, α- and γ-tocopherol were analyzed as described by Sattler et al.^19^, and ubiquinone and ubiquinol as described elsewhere^20^.

Plasma potassium, calcium, magnesium, albumin, fibrinogen, Factor V and Factor VIII were quantified by a medical laboratory (Cerballiance, Paris, France).

### Plasma oxygen reduction rate quantification

Plasma oxygen consumption rate was measured with an oximeter (Oroboros O2k-FluoRespirometer). Immediately after blood collection, samples were centrifuged, and plasma fractions were loaded in closed cuves (2 mL). Oxygen consumption fluxes were assessed when reaching constant values. Experiments were conducted with fresh plasma and after oxidation (exposure to atmospheric air: at least 30 min on a rotator mixer).

### Mitochondria study

*Imaging.* Mitochondria were immunolabeled with anti-SDHA antibody (ab14715, Abcam) and a conjugated Alexa Fluor-568 (2,124,366, Invitrogen); nuclei with DAPI. Cell imaging was performed with a confocal microscope (Leica DM5500 TCS SPE).

*Flow cytometry*. Cells were resuspended in PBS + 2 mM EDTA, labeled with 100 nM TMRM (T5428, Sigma-Aldrich) and analyzed with FACSCcalibur (BD Biosciences). Data were quantified with the FlowJo software (FlowJo, LLC).

### Guinea pig plasma analysis

3-week Dunkin–Hartley guinea pigs (Charles River) were fed for fifteen days with a standard diet (400 mg ascorbate/kg, Safediet ref. 106) or an ascorbate-deficient diet (< 50 mg ascorbate/kg). Blood samples were collected in Hypoxytubes; plasma ascorbate concentration and pO_2_ were determined as described above. Procedure approved by the Institut Pasteur ethics committee (auth. n°190127).

### Statistics

Data were analyzed with the Prism 8 software (GraphPad). ANOVA or Student T-test were performed to analyze the different datasets.

## Supplementary information

Supplementary information
