# Supplementary material for: Ascorbate maintains a low plasma oxygen level
Source: Sci Rep. 2020 Jun 30;10:10659. doi: 10.1038/s41598-020-67778-w (PMC7326906; doi:10.1038/s41598-020-67778-w)
Supplement: Supplementary file 1 — Supplementary information [file 41598_2020_67778_MOESM1_ESM.pdf]

Supplementary informations

Ascorbate maintains a low plasma oxygen level

Authors

Louise Injarabian[1,2], Marc Scherlinger[3], Anne Devin[2], Stéphane Ransac[2], Jens Lykkesfeldt[4], Benoit S Marteyn[1,5,6*]

Injarabian L et al, Supplementary Figure 1

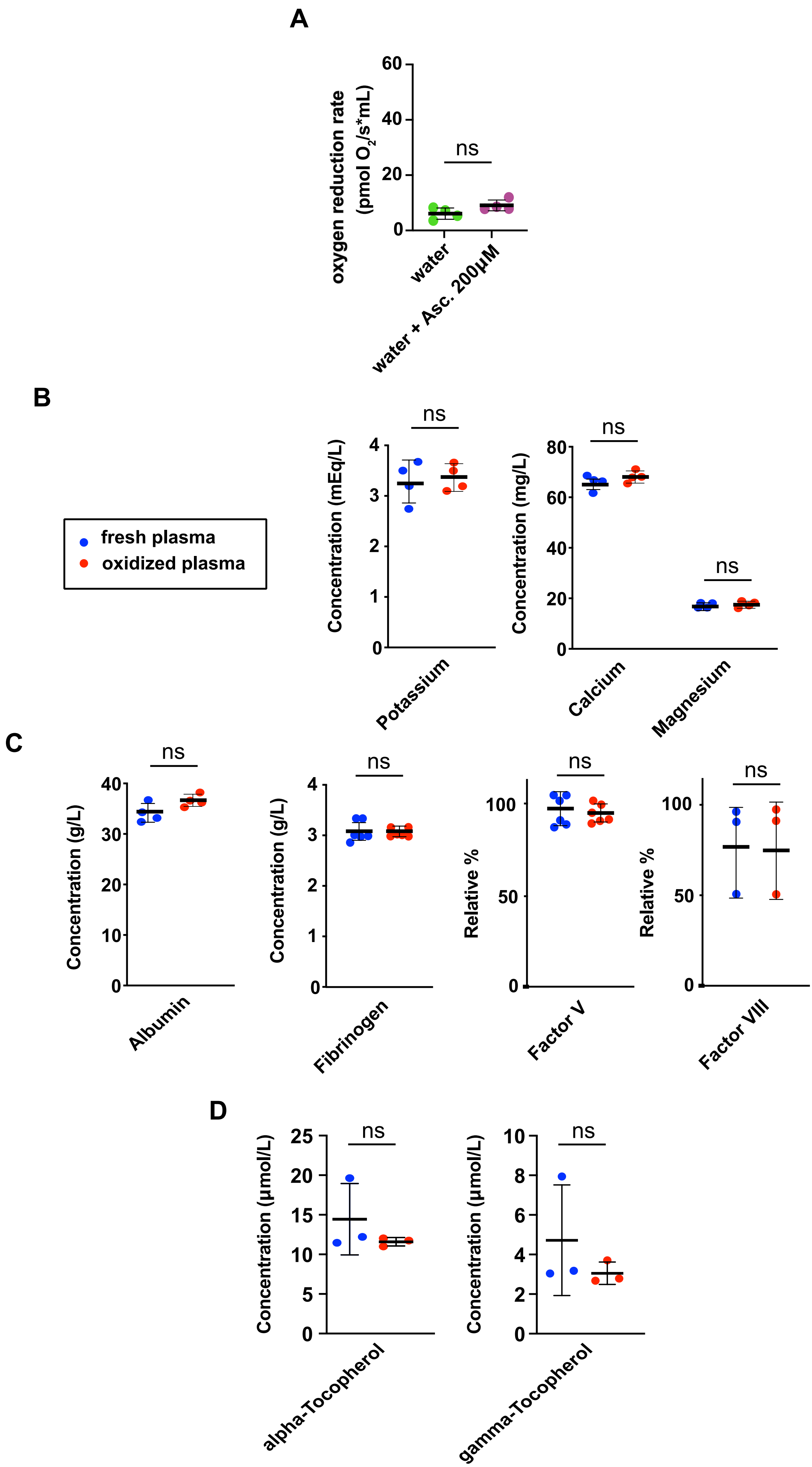

Injarabian L et al, Supplementary Figure 2

A

Tukey's multiple comparisons test	Mean Diff.	95.00% CI of diff.	Significant?	Summary	Adjusted P value
Granulocytes vs. Monocytes	-107,3	-397.5 to 182.8	No	ns	0,7627
Granulocytes vs. Lymphocytes	35,33	-254.8 to 325.5	No	ns	0,9945
Granulocytes vs. Hep-G2	-1468	-1739 to -1197	Yes	****	<0.0001
Granulocytes vs. HEK293T	-834,2	-1106 to -562.8	Yes	****	<0.0001
Monocytes vs. Lymphocytes	142,7	-147.5 to 432.8	No	ns	0,5426
Monocytes vs. Hep-G2	-1361	-1632 to -1089	Yes	****	<0.0001
Monocytes vs. HEK293T	-726,8	-998.2 to -455.5	Yes	****	<0.0001
Lymphocytes vs. Hep-G2	-1503	-1775 to -1232	Yes	****	<0.0001
Lymphocytes vs. HEK293T	-869,5	-1141 to -598.1	Yes	****	<0.0001
Hep-G2 vs. HEK293T	633,8	382.5 to 885.0	Yes	****	<0.0001

B

Test details	Mean 1	Mean 2	Mean Diff.	SE of diff.	n1	n2		DF
Granulocytes vs. Monocytes	153,3	q 260,7	-107,3	91,02	3	3	1,67	12
Granulocytes vs. Lymphocytes	153,3	118	35,33	91,02	3	3	0,55	12
Granulocytes vs. Hep-G2	153,3	1621	-1468	85,14	3	4	24,4	12
Granulocytes vs. HEK293T	153,3	987,5	-834,2	85,14	3	4	13,9	12
Monocytes vs. Lymphocytes	260,7	118	142,7	91,02	3	3	2,22	12
Monocytes vs. Hep-G2	260,7	1621	-1361	85,14	3	4	22,6	12
Monocytes vs. HEK293T	260,7	987,5	-726,8	85,14	3	4	12,1	12
Lymphocytes vs. Hep-G2	118	1621	-1503	85,14	3	4	25	12
Lymphocytes vs. HEK293T	118	987,5	-869,5	85,14	3	4	14,4	12
HepG2 vs. HEK293T	1621	987,5	633,8	78,82	4	4	11,4	12

Supplementary Figure 1

(A) Water oxygen reduction rates was quantified, with and without ascorbate supplementation (200 μM), as described in Fig. 1D-E. Results are expressed as Mean ± S.D.; ‘ns’ indicates $p>0.05$, $n=4$. **(B)** Indicated plasma components (salts, proteins, tocopherol) concentration was determined by a clinical laboratory on fresh plasma samples and after oxidation. Results are expressed as Mean ± S.D.; ‘ns’ indicates $p>0.05$, $n>3$.

Supplementary Figure 2

(A) Results of ANOVA statistical analysis (Tukey’s multiple comparisons test) of TMRM MFI between granulocytes, monocytes, lymphocytes, HepG2 and HEK293T cells (Figure 2D), representing mean difference, 95% confidence interval of the difference, significance, summary and adjusted p-value. **(B)** Details of ANOVA statistical analysis test. **(C)** TMRM MFI in HepG2 and HEK293T cells exposed to atmospheric oxygen (21% O_2) or anoxia (0% O_2) for 24h at 37°C measured with flow cytometry. **(D)** Representation of TMRM profiles of HepG2 and HEK293T cells exposed or not to atmospheric oxygen (21%).
